# The missing voices in global health storytelling

**DOI:** 10.1371/journal.pgph.0003307

**Published:** 2024-07-30

**Authors:** Tiffany Nassiri-Ansari, Anila Jose, Sharifah Khadijah Syed Razif, Emma L. M. Rhule

**Affiliations:** United Nations University–International Institute for Global Health, Kuala Lumpur, Malaysia; PLOS: Public Library of Science, UNITED STATES

In this time of not just poly- but perma-crises, the need for global solutions to global challenges is more apparent than ever. Effective collaboration requires a foundation of mutual trust and respect between individuals, organisations, and nations. However, this foundation is challenged by the colonial legacies that permeate present-day systems and structures and is further eroded by neocolonialism. Thus, in seeking to interrogate barriers to collective action, we must confront not only historical injustices but also the enduring effects of coloniality.

The (re)emergence of de/anti-colonial approaches in global health has brought to the fore analytical tools and languages to identify and interrogate power asymmetries, exclusionary structures, and systemic marginalisation of ways of knowing, being, and doing [1]. Across the contemporary global health ecosystem, scrutiny has been applied to a range of issues, including how funders select grantees and disburse resources [2], how health research, financing, and policy agendas are set at country-level and by whom [3], and who researchers choose to partner with and why [4].

Global health journals–and publishing, more broadly–have not escaped unscathed. From the explicitly colonial roots of journals such as *The Lancet* [5] to exclusionary structures such as exorbitant article processing charges [6] and a bias towards English proficiency [7], global health publishing is one of many domains in which the coloniality of knowledge and continued amplification of high-income country (HIC) voices remains an active force.

**Fig 1:**
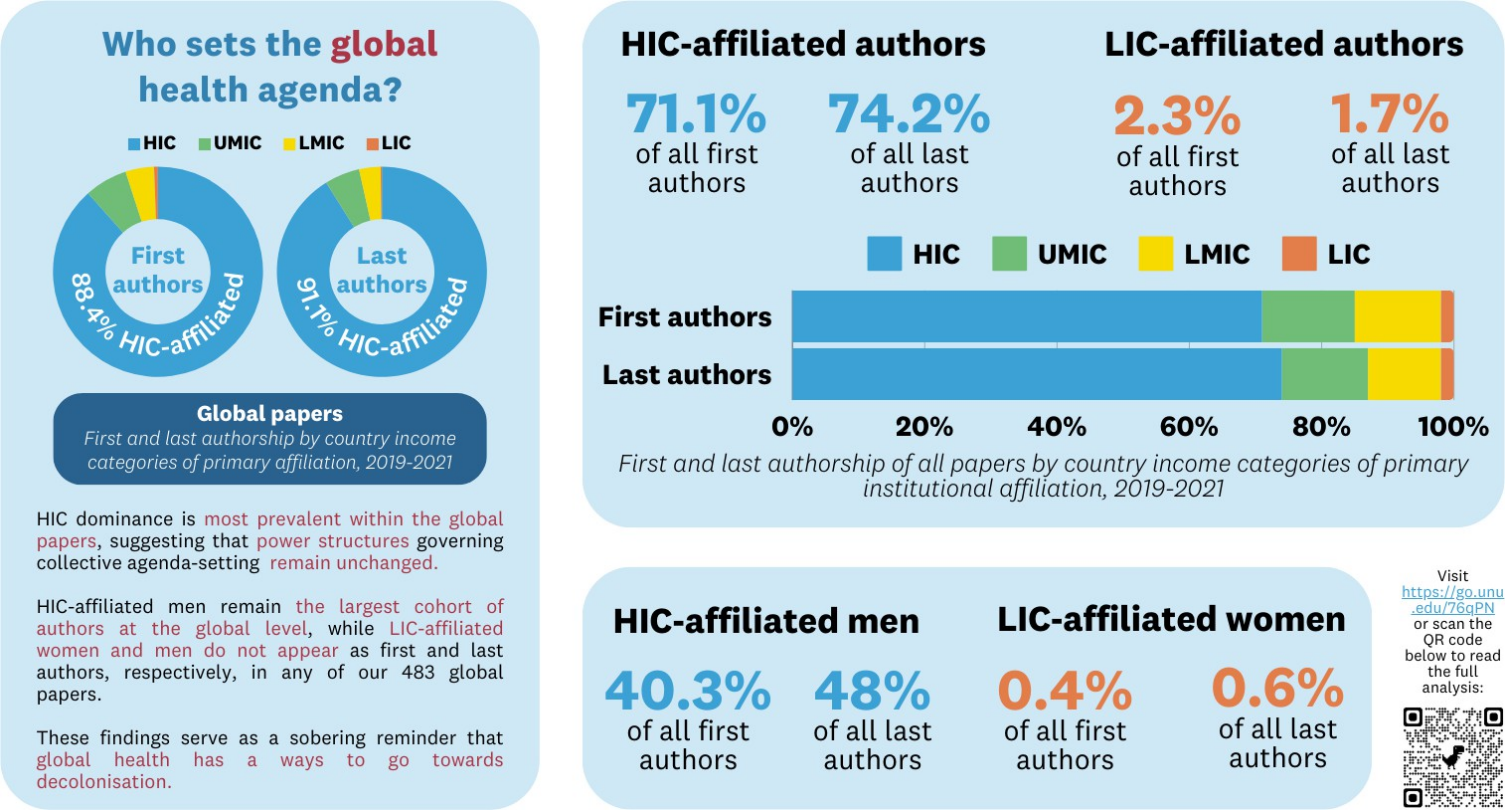
Key findings from our analysis of authorship trends across 1,269 articles.

In a recent analysis of global health authorship patterns [8], we investigate how colonial notions of who we consider experts continue to permeate knowledge production and, accordingly, who is afforded the opportunity to become a global health storyteller [9]. Working with a purposively selected sample of 12 journals encompassing medicine, global health, and infectious diseases, we identified up to 40 eligible articles per year from 2019–2021 for analysis. The inclusion criteria included: primary/secondary research or non-research articles involving at least one LMIC; a focus on human health; and publication in the main journal with named authors visible during title/abstract review. Across our dataset of 1,269 articles, we find a persistent domination of prestigious authorial positions, namely first (71.1%) and last (74.2%) authors, by authors affiliated with institutions in high-income countries (HICs). Within the same dataset, we see the intersecting nature of coloniality as both a racialised and gendered project [1] on full display as men affiliated with HIC institutions form the single largest cohort of both first (40.3%) and last (48%) authors; this cohort unequivocally eclipses that of women affiliated with institutions in low-income countries (LIC), who form the smallest cohort with a representation rate of 0.4% of all first authors and 0.6% of all last authors (see Fig 1).

Looking specifically at papers focused on a single low- or middle-income country, we found a slightly less (but nonetheless) disproportionate allocation of authorship; of the 470 papers in our dataset focused on low-and-middle-income countries (LMICs), 47.2% of first authors and 41.5% of last authors were affiliated with an in-country institution. Whilst still untenable for those of us who champion local knowledge production and ownership, this stands in stark contrast to the prevalent and persistent HIC domination of first and last authorship for multi-country (79.8% and 81.5%, respectively) and global (88.4% and 91.1%, respectively) papers.

We acknowledge and applaud the positive changes that are unfolding within global health publishing, such as the adoption of reflexivity statements [10], the push for more inclusive and transparent peer review processes [11], and even the creation of entirely new journals set up with the explicit goal of addressing these power asymmetries [11].

However, the disparity between marked improvements at the national level and minimal changes at the multi-country and global levels provides insight into the resistance to power redistribution that persists within global health. As long as the criteria for and incentive structures aligned to authorship remain unchanged, change at the scale and speed required will remain elusive. First and last authorship remain highly coveted, with the power to make or break careers [12, 13], leading to situations in which the onus is placed upon individuals to challenge academic hierarchies and incentive structures rather than institutionalising enabling environments that ensure the creators of knowledge are rightfully acknowledged as owners. English remains the dominant language of major global health journals, creating significant barriers to entry for the majority of the world [7].Perhaps most crucially, the ways in which we are willing to identify and validate knowledge remain frustratingly limited, perpetuating an epistemic conformity engineered by colonial powers to ensure that a very limited group of people, adhering to an even more limited set of criteria, qualify as experts.

As abysmal as they are, our findings should come as no surprise to anyone well-versed with the barriers of global health publishing. They serve only as a reminder that incremental changes, whilst valuable in the immediate present, do not necessarily disrupt the systems and structures of coloniality working quietly in the background. Whilst many have rightly pointed out that one way to redistribute power within global health publishing would be to seek alternative avenues of knowledge dissemination [14], we maintain that peer-reviewed journals “remain crucial actors within the global health evidence base due to their power to confer legitimacy upon knowledge” [9] and must therefore be reformed to challenge narrow colonial notions of evidence and expertise.

The increasingly globalised nature of the challenges facing us today demands that we prioritise global, cross-sectoral solutions rather than the reification and imposition of a single way of knowing, being, and doing under the guise of ‘universalism’, as has long been a hallmark of coloniality [15]. However, how global can our solutions be if the evidence bases that inform those solutions remain limited to the point of being exclusionary? Significant changes are urgently needed to restore epistemic pluralism, foster mutual trust and respect, and facilitate collective and collaborative action towards truly global solutions. We hope our findings will contribute to a sustained and relentless demand for systemic and structural change–because if the state of the world today has taught us anything, it is that the status quo is part of the problem, not the solution.
